# Behavioral Characteristics of Children with High Functioning Pervasive Developmental Disorders during a Game

**DOI:** 10.2188/jea.JE20090178

**Published:** 2010-03-05

**Authors:** Hideo Kawaguchi, Bonko Murakami, Masatoshi Kawai

**Affiliations:** 1 Faculty of Life Sciences, Toyo University, Itakura, Gunma, Japan; 2 Research Institute of Science and Technology for Society, Japan Science and Technology Agency, Tokyo, Japan; 3 Center for the Study of Child Development, Institute for Education, Mukogawa Women’s University, Nishinomiya, Hyogo, Japan

**Keywords:** sociability, behavior, developmental disorders, head motion, gaze direction, motion capture system

## Abstract

**Background:**

To evaluate children’s sociability through their behavior, we compared the motion features of children with high functioning pervasive developmental disorders (HFPDD) and typical development (TD) during a game. We selected ‘Jenga’ as the game because this is an interactive game played by two people.

**Methods:**

We observed the behavior of 7 children with HFPDD and 10 children with TD. An optical motion capture system was used to follow the movement of 3-dimensional position markers attached to caps worn by the players.

**Results:**

The range of head motion of the children with HFPDD was narrower than that of the control group, especially in the X-axis direction (perpendicular to the line connecting the two players). In each game, we calculated the range of motion in the X-axis of each child and divided that figure by the matched adult player’s range. The average ratios of children with HFPDD and TD were 0.64 and 0.89 (number of games are 61 and 18), and the difference of these two ratios is significant (*P* < 0.001).

**Conclusions:**

This ratio has sensitivity to identify HFPDD children and could be useful in their child care.

## INTRODUCTION

Sociability is a person’s ability to get along with others, an indispensible skill for social life.^1^^,^^2^ In recent years society of leading industrialized nations has changed in many ways. The increasing pace of technological development has created an information-driven society, a dwindling birthrate, and an ageing populace. The importance of sociability in dealing with such rapid changes to our living and social environment is increasing, especially our children. The “development of sociability” is the main target of our research project, “Identification of Factors Affecting Cognitive and Behavioral Development of Children in Japan Based on a Cohort Study” conducted by Japan Science and Technology Agency.^3^ While sociability can be conceived as higher brain function based on verbal and non-verbal communicative ability,^4^^–^^6^ in many cases the neural basis and developmental terms of the process of acquiring this ability are poorly understood.^7^^–^^10^ As the first step towards identifying the acquisition processes of sociability we aim to develop a method for the quantification of sociability.

We attempted to derive a behavioral indicator connected to sociability based on comparing the behavioral characteristics of two groups of children, children who are socially challenged (with high functioning pervasive developmental disorders (HFPDD)) and those who are not (children with typical development (TD)).^11^^,^^12^

**Figure 1.**
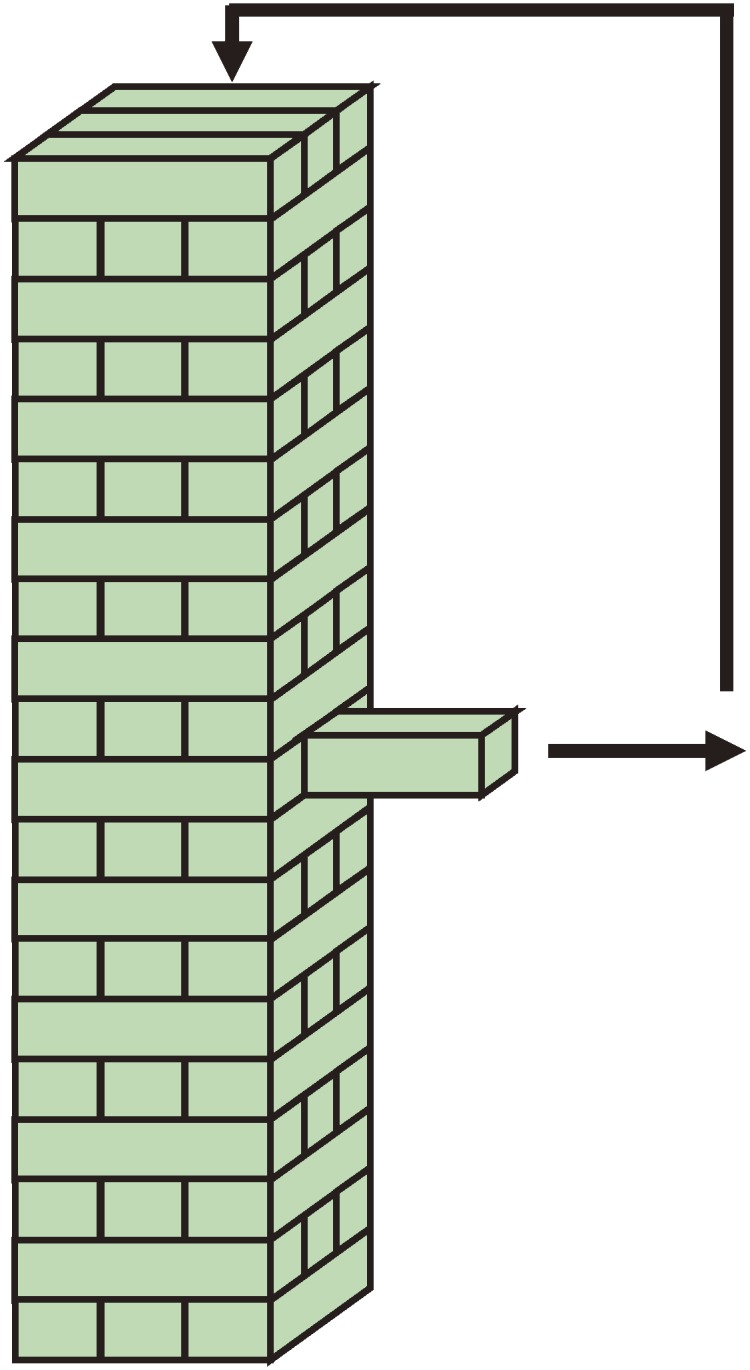
Schema of Jenga. Jenga is a balancing game where players remove wooden blocks form a tower and place them on top of the tower. (Jenga: “to build” in Swahili)

A game played between two players was used to create social interaction that would allow us to gain an understanding of sociability.^13^^,^^14^ We selected ‘Jenga’, a balancing game using stacked wooden blocks (Figure 1).^15^ The same adult examiner played through all games of Jenga. The examiner’s opponents were children with HFPDD and children with TD. In Jenga, a player searched for a wooden block to remove, and an opponent almost gazed at the same block (or the player’s fingers). This process should be “joint attention”^16^^,^^17^, and the process would trigger opponent’s behavior gazing the lateral sides of Jenga tower of wooden blocks. This must be the social-interactive behavior which we can measure physically.

We attempted to derive the social implications of differences in behavioral characteristics observed between the HFPDD children and the TD children during the game. We also coded children’s gaze directions, considered to be an important signal socially,^18^^,^^19^ using video data and compared them with behavioral data. A behavioral indicator connected to sociability will be useful as a quantitative method for evaluating sociability.

## METHODS

### Participants

Subjects in the study consisted of participants from whom written informed consent had been obtained, based on a consent acquisition protocol approved by the ethics committee of Mukogawa Women’s University. Of the subjects, seven were children with HFPDD (from 9 to 12 years of age) and ten were children with TD (from 6 to 11 years of age). All subjects were right-handed except one HFPDD child who was left-handed. All children with HFPDD who were subjects in the study had received a definite diagnosis for their condition, and at the time were undergoing special education as therapy.

The same healthy, adult, right-handed female examiner participated in all the games.

During measurements the examiner followed a prescribed procedure in a given order. The examiner would lead the HFPDD child or the TD child to the observation room. On entering the observation room the examiner would ensure the child was wearing the cap with attached infrared reflective markers. The examiner explained the rules of Jenga to the child, a period of practice play was held, and then the actual game of Jenga was played.

### Game

The game of Jenga (TOMY Co., Ltd.) was played between one examiner and one participant. The Jenga game was placed on a standard conference table, with the examiner and participant sat at opposite sides of the table while playing the game.

The game rules were as follows: Players take turns to remove a wooden block from the tower and place it on top of the tower. A player loses if they cause the blocks to fall on their turn or they are unable to remove a block. There is a 15-minute time limit on playing the game. The game is continued until the time limit has been reached; no matter how many times a player loses. An unfinished game is considered a draw.

Turns of the game exchanged according to the following rules. The turn returns to the player after the opposing player is instructed to return their hand to their lap, and the turn begins with the player the moment the player’s hand lifts above the edge of the player’s side of the table. After placing a wooden block on top of the tower, the player’s turn ends the moment the player’s hand reaches below the edge of the table on the player’s side. Turns taken by the opposition player are identical to those taken by the player. Periods may occur when players’ turns overlap (false-start play), or when several seconds of no action elapses between turns.

### Observation room

**Figure 2.**
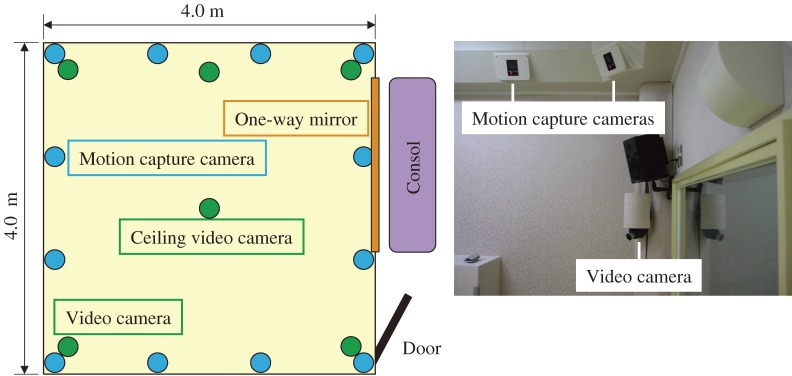
Arrangement of the observation room

Figure 2 shows the arrangement of the observation room. The room is an adapted soundproof room (AVITECS, Yamaha Corporation) of four by four meters square, and provides a soundproofed environment. The soundproofing is to ensure the concentration of children participating is not distracted by ambient noise during the game. Six CCD video cameras, two microphones, and an optical motion capture system set up inside the observation room recorded the state of social interaction of the two players during the game. Four CCD cameras were installed approximately two meters from the floor at each corner of the room. One CCD camera was installed in the center of the ceiling and looked down on the player’s movements. One more CCD camera was installed near the wall at near-to table height, perpendicular from the line joining the two seated players, and recorded movement of the players from the side. An operator adjusted the direction and zoom controls of the CCD video cameras to ensure the whole of the examiner’s and the participant’s body was within the field of view of each camera. The operator was placed behind a one-way mirror and observed the behavior of the examiner and the participant through the mirror. Both of the microphones were installed at fixed positions on the ceiling of the room. The motion capture system is described below.

### Motion capture system

The state of interaction of the players during the game was recorded using an optical motion capture system (Qualysis Inc., Sweden). The system consisted of twelve infrared cameras installed close to the ceiling of the observation room (Figure 2) recording the players’ behaviors during the game by detecting the spatial position of several reflective markers attached to caps worn by both players. The twelve cameras were installed equidistant from each other in a configuration of four cameras along the plane of each wall (output from each of the four corner cameras was shared between walls). Infrared light was provided by an LED array strobe mounted on the front of each camera. A sampling frequency of 30 Hz was chosen to provide stable data transfer and processing over a hypothetical period of almost half hour of continuous measurement. The median trajectory of each of three or four visible reflective markers was used by the system to draw the movement of each player’s head during the game.

### Gaze direction coding procedure

Coding of the HFPDD child’s and the TD child’s gaze direction was carried out using video-images recorded by the six CCD cameras. The number of games coded are 30 and 18, respectively. Gaze direction is considered to be a socially important signal allowing one to understand the subject’s immediate object of interest. Six categories of gaze direction are as follows: Examiner’s fingers, tower/blocks, examiner’s eyes, examiner’s body, participant’s body, others. Sampling periods of 250 ms were taken for coding. All coders were previously trained with test coding images until they reached a rate of agreement of 90% or more. All data was coded by two coders and data confirmed for a rate of agreement between the coders of 85% or more. 54% of the coding data was then worked to an agreement rate of 100% on discussion between two coders, where this data became the final data.

### Analyses

The maximum value and minimum value of the trajectory of motion of each player were obtained along each of the X-axis, Y-axis, and Z-axis of movement, and the difference (range of movement) between those values was determined (ΔX, ΔY and ΔZ). To avoid overestimation of single sudden movements, on each axis the average value was taken of 100 pieces of data extracted in descending order from the highest value taken on that axis, and the resulting value defined as the maximum. Minimum values were estimated similarly. The range of head motion of the HFPDD child participant and the TD child participant along the X-axis was denoted ΔXp, and was compared to the equivalent range of head motion of the examiner (ΔXe) to calculate the participant’s range of movement relative to the examiner (Equation [Disp-formula e01]). The distribution of values of relative range of head motion was then determined. The range of movement of the participant relative to the examiner was calculated in a similar way during the participant’s turns, and during the examiner’s turns (Equations [Disp-formula e02] and [Disp-formula e03]), and the distribution of the values compared. Identical operations were performed for Y-axis and Z-axis data.

ΔX-ratio=ΔXp/ΔXe (1)

$$\begin{array}{l} \Delta \mathrm{X-ratio}(\mathrm{participant}’\mathrm{s turns})\\ =\Delta \mathrm{Xp}(\mathrm{participant}’\text{s turns})/\Delta \mathrm{Xe}(\mathrm{participant}’\mathrm{s turns}) \end{array}$$ (2)

$$\begin{array}{l} \Delta \mathrm{X-ratio}(\mathrm{examiner}’\mathrm{s turns})\\ =\Delta \mathrm{Xp}(\mathrm{examiner}’\mathrm{s turns})/\Delta \mathrm{Xe}(\mathrm{examiner}’\mathrm{s turns}) \end{array}$$ (3)

## RESULTS

**Figure 3.**
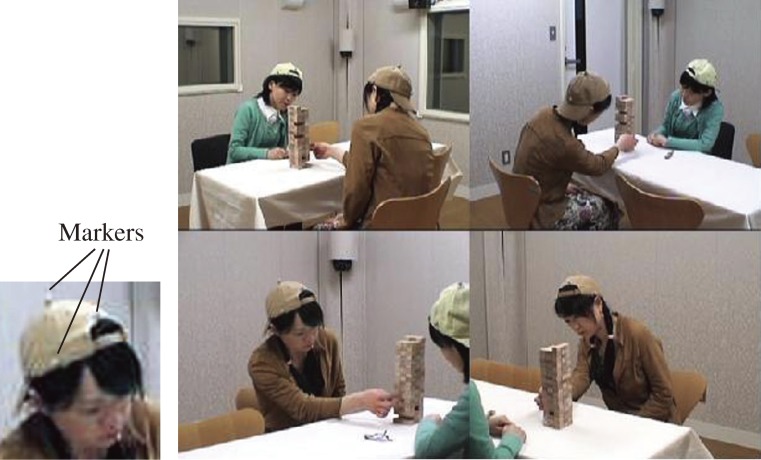
Typical interactive motions of players during Jenga. This is a simulated experiment conducted with adults.

Figure 3 shows a view of the observation room during measurement (photograph of a simulated experiment between two adults). All games were conducted with both players sitting in their seats at all times, except one occasion during a game when a HFPDD child left the chair to play on the floor. The data from that occasion was disregarded, while all other motion capture data was used as obtained.

Players’ turns overlap (false-start play) occurred for some of the time (turn starting too early by one or two seconds) in spite of the examiner instructing participants not to do so each time it occurred. Empty periods of no action also occurred between turns though of no more than a few seconds on almost all occasions.

**Figure 4.**
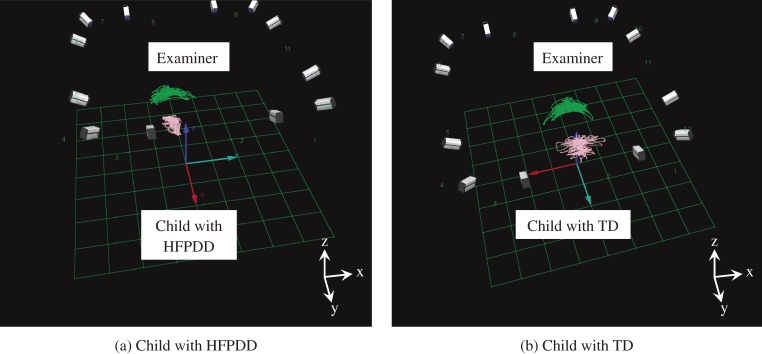
Tracking head motion

**Figure 5.**
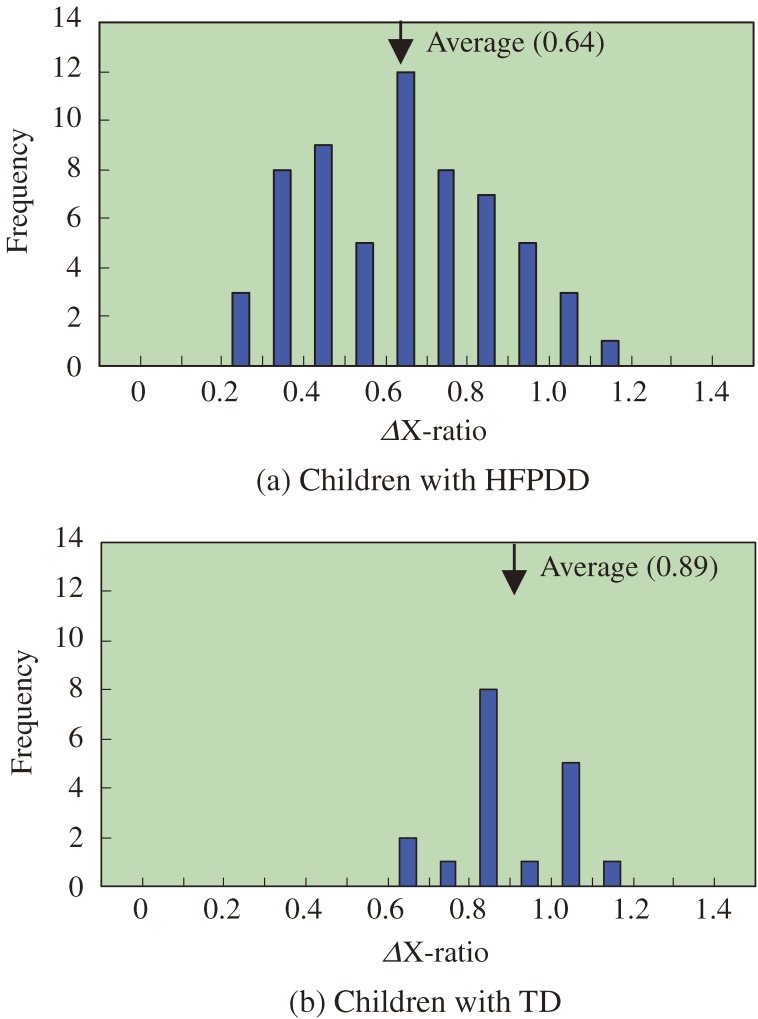
Ratio of Head Motion Range in the X-axis Direction. Each of the seven HFPDD children carried out five experiments in total with each child undertaking one experiment per month. Matches of Jenga played were 61 in case of HFPDD children. While, all the ten TD children undertook one experiment each, then matches of Jenga played were 18.

Each of the seven HFPDD children carried out five experiments in total (matches of Jenga played: 61), with each child undertaking one experiment per month. All ten TD children undertook one experiment each (matches of Jenga played: 18). From the results we ascertained there was a strong tendency for the HFPDD children’s range of head motion along the X-axis to be narrower than the examiner during the Jenga game (Figure 4). On comparing values of ΔX-ratio, an indicator of relative movement based on the examiner’s motion, the ΔX-ratio mean for HFPDD children was 0.60 with a variance of 0.046, while for TD children the ΔX-ratio mean was 0.90 with a variance of 0.021 (Figure 5). Mean values for the groups of HFPDD and TD children measured were found significantly different (*P* < 0.001). It may be possible to indentify between children with HFPDD and children with TD using ΔX-ratio, an indicator of relative head movement along the X-axis.

There was no significant difference in mean values for range of the examiner’s head motion along the X-axis between playing with the HFPDD children and the TD children. No significant difference was also found in Y-axis and Z-axis movement of all players.

**Figure 6.**
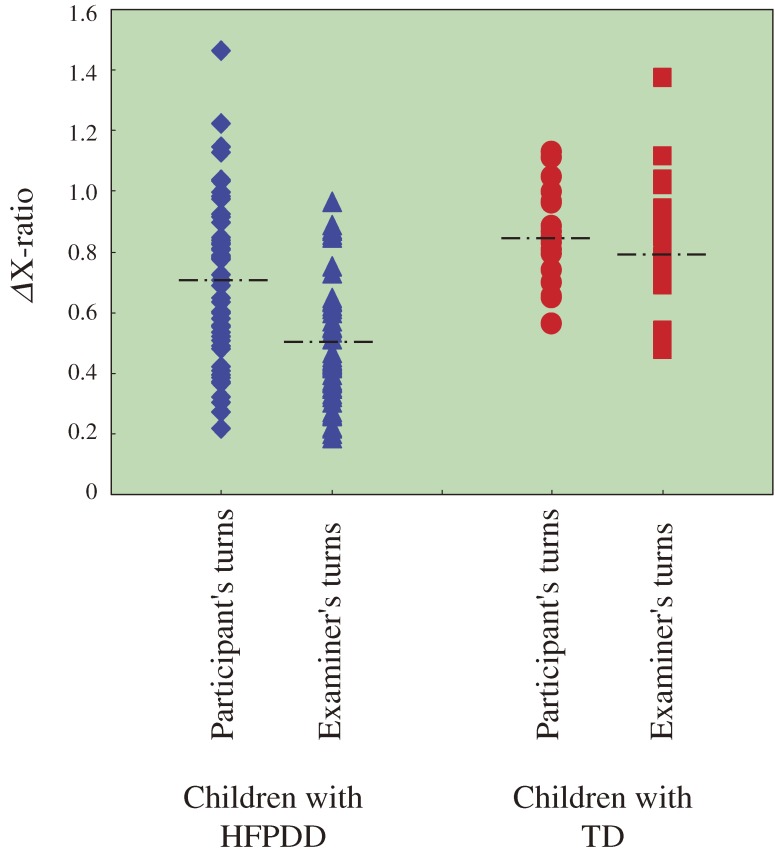
ΔX-ratio in cases of participant’s and examiner’s turns

The difference between values of ΔX-ratio between the period of time the participant is taking a turn, and the period of time the examiner is taking a turn, are compared for children with HFPDD and children with TD (Figure 6). The difference in values of ΔX-ratio between the participant’s turns and the examiner’s turns is clearly greater among HFPDD children. While no significant difference was seen for TD children, among HFPDD children the difference was found to be significant (*P* < 0.001).

Both the HFPDD children and TD children naturally gazed the lateral sides of the tower during their turn to find a wooden block to remove. The HFPDD children and the TD children displayed the similar gazing behavior during the participant’s turn when they were searching for a wooden block. In the same way, there was no significant difference among ranges of the examiner’s head motion along the X-axis during the HFPDD children’s turn or the TD children’s turn. Therefore, the reason for the ΔX-ratio being significantly smaller in the HFPDD children is understood to depend on the narrower range of head motion made along the X-axis by the HFPDD children during the examiner’s turn.

**Figure 7.**
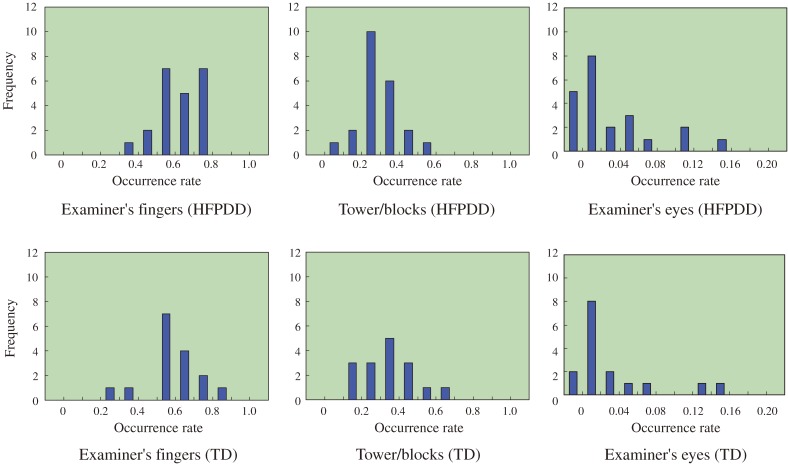
Histograms of time-occurrence rates in major three categories. Major three categories: Examiner’s fingers, tower/blocks, examiner’s eyes.

The above results lead us to believe that the HFPDD children may be less interested in their game opponent, the examiner, than the TD children. We estimated which objects participants were directing their gaze towards, or to what they were paying attention, during the examiner’s turns. Figure 7 shows occurrence rates for the three main categories of gaze direction (examiner’s fingers, tower/blocks, examiner’s eyes). The occurrence rates shown are the integrated proportion of time spent on defined categories of gaze direction arising over the period of experimentation. The results show no significant difference between HFPDD children and TD children in occurrence rates for objects. The occurrence rates show the HFPDD children were directing their gaze towards the examiner’s fingers during the examiner’s turn, and were therefore directing their attention towards the opponent player’s movements when searching for a wooden block to remove.

## DISCUSSION

On video-images taken during the game, the differences in values of ΔX-ratio shown above have been produced by the particularly small side-to-side head motion made by the HFPDD children while gazing the lateral sides of the Jenga tower of wooden blocks. This gazing behavior is thought to be a result of the drawing-in phenomenon of a person’s behavior (entrainment) (Figure 3).^20^ The behavior of someone who is gazing something draws a person into gazing that same thing. This entrainment is speculated to be lesser among HFPDD children based on results that show ΔX-ratio values were significantly smaller for the HFPDD children when compared to the TD children.

This entrainment arises when a subject is inspired by the behavior of another.^21^^,^^22^ Even if the same examiner is present throughout all the experiments, differences in the examiner’s own movements are likely to arise depending on whether they are together with a HFPDD child or a TD child, as differences of behavior exist between HFPDD and TD children. This nested structure exists because of social interactions and mutual relationships. Taking this into account, absolute values of ΔX were not used as an indicator of sociability, instead the ΔX-ratio was used, as an indicator of the behavior of the participant relative to the examiner.

A comparison of different examiners was not conducted by this study. The dependence of ΔX-ratio on the examiner should be investigated in future studies.

A difference was seen between HFPDD children and TD children in range of head motion along the X-axis during the examiner’s turn, though no difference was seen in occurrence rates for gaze direction. This seems to show that while the HFPDD children were as interested as the TD children in the opponent’s (the examiner’s) behavior, the HFPDD children could not transfer this interest into movement as capably as TD children. This result seems to indicate that HFPDD children have problems conveying their interests through movement, as they are not good at non-verbal communication.

As mentioned previously, the difference in range of head motion along the X-axis between HFPDD and TD children during the examiner’s turn can be described as the difference in extent to which the children’s attention has been drawn to the examiner’s motions in searching for and removing a wooden block. This entrainment that follows the examiner’s gaze and objects delineated by the examiner’s fingers gives rise to the joint attention process.^23^^–^^28^ Head motion along the X-axis can therefore be interpreted as movement required to achieve this joint attention while the Jenga tower poses to block the field of view. The Jenga employed in this study may therefore be a useful method for transforming and amplifying the gaze movement known as joint attention into head motion along the X-axis, a physical parameter simple to measure.

Regarding the behavioral characteristics of sociability shown in the oppositional game scenario employed in this study, if the elementary process and significance of such behavior is determined, and a subject’s brain function can be simultaneously measured, we could demonstrate the neural basis for the elementary processes of that behavior.^29^^–^^32^ Using a portable-model optical topography system now makes the unconstrained and noninvasive measurement of brain function possible without disturbing the course of an actual game.^33^^–^^36^

This study treated all head motions obtained during a single experiment as a single trajectory and investigated comparisons made between them. Though a time series analysis of data was not conducted in this study, part of the analysis carried out by this study was of data separated into data arising during the participant’s turn, and data arising during the examiner’s turn. Measurements made by a motion capture system have the advantage of providing a high temporal resolution. Further investigation is needed to make proper use of this potential.

There are two possible strategies when playing the game Jenga. One strategy is to play for mutual enjoyment and allow the game to continue for as long as possible, while the other strategy is to aim to simply win the game. While no specific instructions were given to the examiner or the participants in this study regarding the strategies above, either strategy may potentially give rise to different levels of behavior.^37^^,^^38^ For example, there is huge potential for the competitive strategy to dilute social interactions during the game, as one person may play the game while ignoring the opponent’s attitude and intent, only concentrating on what oneself is doing and ignoring the opponent’s bodily movement. The change in behavior caused by such instructions will be a subject of future investigation.^39^

Having established a method of quantifying sociability, we will proceed to organize and investigate the implementation of this method as a methodology for quantifying sociability where an application is conceived possible, during future long-term cohort studies.

As the motion tracking used in this study is visualized in real time, one example of a beneficial application of this technique is during the medical examination carried out preschool children. The monitoring of the change in behavioral characteristics of a child with developmental disorders over time can be used as direct feedback to care the child.^40^^–^^44^ Furthermore, if the item to be measured is for example narrowed down to the ΔX-ratio, this should allow use of measurement methods simpler than a motion capture system.

The above results of coding gaze direction did not show HFPDD children to not meet the gaze of the examiner, rather they showed the rate of occurrence for this meeting was no different between the HFPDD and the TD children. This experimental fact is in contradiction with the diagnostic criteria for autism given by DSM-IV^45^ of averting the eyes from direct eye contact. A child with HFPDD is creating communicative signals, but the receiver is indicating they cannot recognize the signals. This is similar to the gap mentioned previously between movement along the X-axis and gaze direction of the HFPDD children during the examiner’s turns. Making the assumption that children with developmental disorders are creating communicative signals, if we make efforts to pick up those signals, and educate widely with this objective, we may be able to create a society where children with developmental disorders can live comfortably.
